# Quantitative assessment of bone marrow infiltration and characterization of tumor burden using dual-layer spectral CT in patients with multiple myeloma

**DOI:** 10.2478/raon-2024-0003

**Published:** 2024-01-06

**Authors:** Xing Xiong, Rong Hong, Xu Fan, Zhengmei Hao, Xiaohui Zhang, Yu Zhang, Chunhong Hu

**Affiliations:** Department of Radiology, The First Affiliated Hospital of Soochow University, Suzhou, Jiangsu, China; Department of Clinical Science, Philips Healthcare Greater China, Shanghai, China; Department of Radiology, Dushu Lake Hospital Affiliated to Soochow University, Suzhou, China

**Keywords:** bone marrow, tumor burden, virtual non-calcium, dual energy CT, multiple myeloma

## Abstract

**Background:**

The aim of the study was to evaluate whether virtual calcium subtraction (VNCa) image extracted from dual-layer spectral CT could estimate bone marrow (BM) infiltration with MRI as the reference standard and characterize tumor burden in patients with multiple myeloma (MM).

**Patients and methods:**

Forty-seven patients with newly diagnosed MM were retrospectively enrolled. They had undergone whole-body low-dose dual-layer spectral CT (DLCT) and whole-body MRI within one week. VNCa images with calcium-suppressed (CaSupp) indices ranging from 25 to 95 at an interval of 10 and apparent diffusion coefficient (ADC) maps were quantitatively analyzed on vertebral bodies L1−L5 at the central slice of images. The optimal combination was selected by correlation analysis between CT numbers and ADC values. Then, it was used to characterize tumor burden by correlation analysis and receiver operating characteristic (ROC) curves analysis, including plasma cell infiltration rate (PCIR), high serum-free light chains (SFLC) ratio and the high-risk cytogenetic (HRC) status.

**Results:**

The most significant quantitative correlation between CT numbers of VNCa images and ADC values could be found at CaSupp index 85 for averaged L1−L5 (r = 0.612, p < 0.001). It allowed quantitative evaluation of PCIR (r = 0.835, p < 0.001). It could also anticipate high SFLC ratio and the HRC status with *area under the curve* (*AUC*) of 0.876 and 0.760, respectively.

**Conclusions:**

The VNCa measurements of averaged L1−L5 showed the highest correlation with ADC at CaSupp index 85. It could therefore be used as additional imaging biomarker for non-invasive assessment of tumor burden if ADC is not feasible.

## Introduction

Multiple myeloma (MM) is one of the malignant hematological diseases with monoclonal proliferation of plasma cells which primarily involves bone marrow (BM).^1^ “Myeloma bone disease” forms when malignant proliferation of plasma cells displaces the healthy BM, and then results in activation of osteoclasts and inhibition of osteoblastic activity.^2,3^ The characterization of BM tumor burden has important indications for treatment regimens, treatment response and surveillance. It has been exclusively accomplished by BM biopsy and serologic/urine markers such as plasma cell infiltration rate (PCIR), serum-free light chains (SFLC) ratio, paraproteins (M-protein) in serum/urine and cytogenetic status.^4,5,6^ However, these biomarkers examinations suffer unavoidable deficits such as invasive, painful and expensive.

As first introduced by Durie and Salmon in 1975, conventional radiographic survey of the skeleton was applied to stage MM bone disease.^7^ However, owing to low sensitivity in detecting osteolytic lesions and unable to evaluate therapy response, it calls for more practical techniques to be used. With the development of imaging techniques such as monoenergetic computed tomography (MECT), magnetic resonance imaging (MRI), and fluorodeoxyglucose positron-emission-tomography CT (FDG PET/CT), direct evaluation of BM infiltration has become possible.^8^ MECT is widespread available and economic efficient, so MM patients are commonly first assessed with whole body MECT scans.^9^ The major limitation of MECT is low sensitivity for detecting nonlytic BM infiltration in the axial skeleton, which is more common for MM patients. MRI is confirmed to be “imaging golden standard” for BM infiltration which has proven higher sensitivity in detecting MM lesions than any other modality.^10^ Whereas, it takes long time to accomplish examination for patients which may cause unbearable pain and claustrophobia.^11,12^ FDG PET/CT has been lately recommended to evaluate response and residual activity in treated patients as it could respond to BM changes quickly.^13^ However, the associated radiation and economic cost should be considered.

Dual-layer spectral CT (DLCT) is a novel CT technique with two different detector layers atop each other to absorb different parts of the polychromatic-attenuated X-ray spectrum. It could construct various parameter images e.g., uric acid, iodine, or calcium according to the aim of research retrospectively. Recent studies showed that DLCT, especially virtual non-calcium (VNCa) image, shows significant improvements in comparison to MECT and comparable to FDG PET/CT and MRI in the evaluation of MM.^14,15,16^ Hence, our study had two objectives: firstly, to explore the potential of VNCa image in estimating BM infiltration with MRI as the reference standard in MM patients. Secondly, to identify if VNCa image could characterize tumor burden by correlate with established biomarkers (PCIR, SFLC ratio and cytogenetic status).

## Patients and methods

### Patient characteristics

The study was approved by ethics committee of local institution and the need for written informed consent was waived due to retrospective nature of the study (registration number: 000/2021). All scans were performed for conventional clinical requirements.

We have collected the information of MM patients from 6/2021 to 10/2022 admitted to our institution consecutively. The inclusion criteria were as follows: (1) histologically confirmed diagnosis of MM; (2) the interval between clinical data, whole-body low-dose DLCT and whole-body MRI examination no more than two weeks; (3) received no specific therapy for MM before. The exclusion criteria were as follows: (1) patient’s age below 18 years; (2) no complete clinical data, neither DLCT nor MRI examination; (3) obvious metal or motion artifacts affecting the lumbar vertebral segmentation.

### Imaging acquisition and post-processing

All scans were performed on a commercially available spectral detector DLCT scanner (IQon Spectral CT, Philips Healthcare), following the most recent recommendations of the International Myeloma Working Group (IMWG).^17^ Patients were placed in a head-first supine position. The scan ranges from vertex of the skull to the knees. No contrast agent was given. Scan parameters were as follows: tube voltage, 120 kV; tube current, 70 mAs; collimation, 64×0.625 mm; pitch, 0.990; rotation time, 0.75 s; volumetric computed tomography dose index, 7.4 mGy. Mean dose length product was 1069.2 ± 205.9 mGy*cm. The field of view (FOV) was adjusted depending on patient body volume.

The corresponding MRI examination was performed on a 3.0 T scanner (Magnetic Verio, Siemens Healthcare, Erlangen Germany). The patients were also placed in a head-first supine position. Phased-array surface coils were installed to cover from the head to the upper femur. No contrast medium was given. The protocol parameters were as follows: T2 turbo inversion recovery magnitude (TIRM) sequence [echo time (TE), 84 ms; repetition time (TR), 7110 ms; slice thickness, 5 mm; slice gap, 1.5 mm; FOV, 480 mm] was acquired on the coronal plane from the head to the upper femur. On the same coverage area, axial DWI sequences were acquired using two values (b = 50, 700 s/mm^2^) with the following parameters: TR, 4000 ms; TE, 46 ms; slice thickness, 5 mm; slice gap, 0; FOV, 450 mm.

Post-processing of spectral-based image (SBI) data was performed with the vendor’s software (IntelliSpace Portal Version 11, Philips Healthcare). First, all SBI images were reconstructed in a 512 × 512 matrix, slice thickness 2 mm with an overlap of 1 mm. Then, VNCa images were created from SBI data by exploiting the material specific attenuation of X-rays in different energy levels to simulate each voxels attenuation in Hounsfield units without the calcium-specific contribution. The intelligent post-processing vendor allows calcium suppression in seamlessly adjustable factors. In our study, VNCa images were reconstructed with calcium-suppressed (CaSupp) indices ranging from 25 to 95 in steps of 10. Among them, CaSupp indice 25 means images has minimum visibility of bony structures and 95 means maximum visibility.

### Segmentation of the bone marrow

**FIGURE 1.**
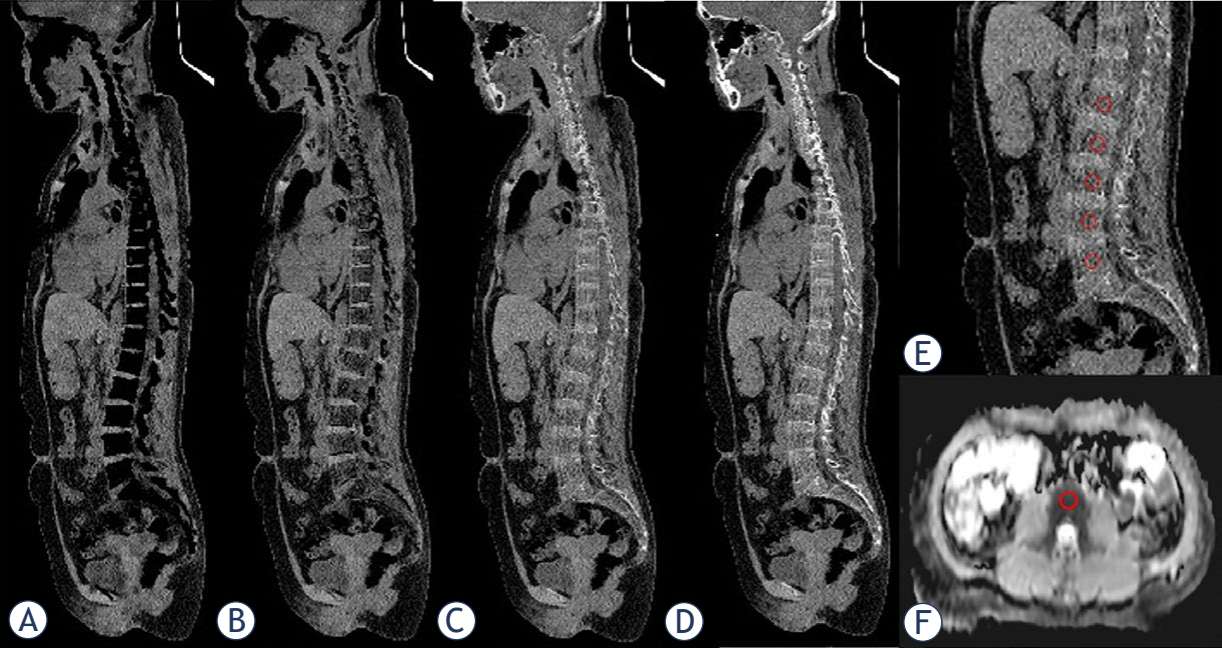
An example of bone marrow (BM) segmentation in multiple myeloma (MM) patient. An oval regions of interests (ROIs) of 100 mm2 was drawn at the central slice of sagittal vertebral bodies L1−L5 in different virtual calcium subtraction (VNCa) images and the corresponding location was manually displayed on the axial apparent diffusion coefficient (ADC) map. **(A)** calcium-suppressed (CaSupp) index 25, **(B)** CaSupp index 55, **(C)** CaSupp index 85, **(D)** CaSupp index95, **(E)** magnification of **(C)**, **(F)** ADC map.

Although the MM lesions were scattered, it involved typical location such as lumbar vertebra, pelvis and ribs. So, we chose to focus on L1−L5 due to the large size of those vertebrae with maximized reliable measurement, typical sites of BM infiltration and minimally affected by the intrauterine device.^18,19^ Using the same software, regions of interests (ROIs) were positioned manually in the sagittal vertebral bodies L1−L5 to measure the respective CT numbers and basivertebral vein was avoided from the ROIs. Since the lumbar vertebra were wide, a standard circular ROI which size set to 100 mm^2^ was placed at the central slice. To ensure comparability, ROIs were copied between different CaSupp indices. At the same time, the corresponding location was contoured manually on the axial apparent diffusion coefficient (ADC) map (Figure 1). The images were analyzed by two radiologists with more than 5 years of experience who were blinded to any patient information. The intraclass correlation coefficient (ICC) was calculated for determining the interrater reliability of the quantitative assessment. The final CT number and ADC values were averaged. The analysis of VNCa and ADC images was conducted for 10 min per person.

### Assessment of established biomarkers

PCIR was obtained through BM biopsy on the wing of ilium and assessed by our in-house pathologists. Immunoturbidimetry was used to detect the expression levels of SFLC kappa and lambda. SFLC ratio were classified as high (<0.01 or >100) or low (0.01−100) according to the IMWG criteria and the practical experience of our institution.^20,21^ Cytogenetic status was performed by fluorescence in situ hybridization (FISH) in interphase cells to overcome the problem of karyotyping. MM patients were divided into high-risk cytogenetic (HRC) and standard risk cytogenetic (SRC) groups on the basis of FISH results. Patients who presented with any of the following cytogenetic abnormalities (CAs) were categorized into the HRC group: del(17p), t(4;14), t(14;16), t (14;20), gain(1p), or p53 mutation. Other MM patients were allocated into the SRC group.

### Statistic assessment

Statistical analysis was performed by either SPSS 22.0 software (Chicago, IL, USA) or MedCalc statistical software version 16.4.3 (Ostend, Belgium). Correlations between different VNCa CT numbers (combined L1−L5 with different CaSupp indices) and ADC values were calculated. Since VNCa CT numbers and ADC values were normally distributed, Pearson’s correlation analysis was applied. Then the optimal combination was used to characterize PCIR by correlation analysis and receiver operating characteristic curves (ROC) analysis was carried out to predict binary outcomes “high SFLC ratio” and “HRC status”. Statistical significance was defined as p ≤ 0.05.

## Results

### Patient characteristics

**FIGURE 2.**
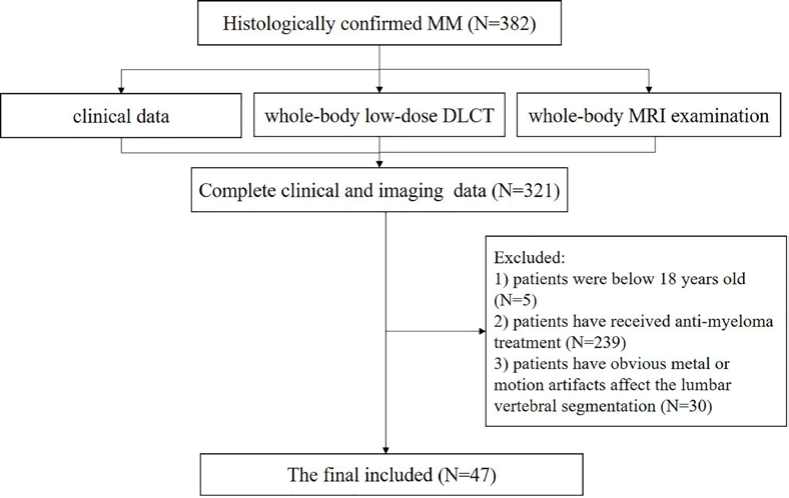
Flow chart of patients’ selection. DLCT = low-dose dual-layer spectral CT; MM = multiple myeloma

A total of 382 MM patients were admitted at the hematology center in our institution for whole-body DLCT. Of these, 5 patients had to be excluded because they were under 18 years old. 239 patients had to be excluded because they have received anti-myeloma treatment. 61 patients had no complete clinical data, neither DLCT nor MRI examination. Another 30 patients had obvious metal or motion artifacts that affected the lumbar vertebral segmentation. Consequently, 47 MM patients were included. Enrollment results of MM patients after exclusion were shown in the Figure 2. The average interval between the clinical examination and DLCT scan was 10 days [interquartile range 3.0−13.5 days]. The clinical characteristics are shown in Table 1. The interrater reliability of all quantitative measurements was very high with ICC ranged from 0.824−0.970.

**TABLE 1.** Patient characteristics

**Characteristics**	**n**
Age^*^	57.9 ± 8.1
Sex^#^	
Males	26 (55.3%)
Females	21 (44.7%)
Myeloma subtypes^#^	
IgG	32 (68.1%)
IgA	10 (21.3%)
Light chain	5 (10.6%)
Plasma cell infiltration ratio obtained from the wing of ilium^*^	0.54 ± 0.30
Kappa/lambda SFLC ratio^#^	
High (< 0.01 or > 100)	27 (57.4%)
Low (0.01−100)	20 (42.6%)
Cytogenetic status^#^	
HRC	26 (55.3%)
SRC	21 (44.7%)

* represented as Mean ± SD;

# represented as number (percentage)

HRC = high-risk cytogenetic; SD = Standard deviation; SFLC = serum-free light chains; SRC = standard risk cytogenetics

### Correlation analysis

**FIGURE 3.**
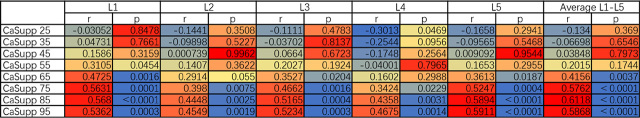
Heat map of Pearson’s correlation r and p value between CT numbers (combined different calcium-suppressed [CaSupp] indices with measured locations) and apparent diffusion coefficient (ADC) values.

1880 and 235 ROIs were derived from VNCa images for different CaSupp indices and ADC maps, respectively. Table 2 shows the mean ADC values and CT numbers (combination of different vertebral bodies and CaSupp indices). Regardless of the measured location, CT numbers in VNCa images at CaSupp indices from 75 to 95 were significantly correlated with ADC (Pearson’s r ranges from 0.342−0.612, with all p < 0.05). Inversely, CT numbers in VNCa images at CaSupp indices from 35 to 45 showed no correlation with ADC for all locations. The highest correlation of VNCa-CT numbers and ADC values was averaged L1−L5 at CaSupp indices 85 (Pearson’s r = 0.612, p < 0.001). Figure 3 provides the statistical results regarding the correlation between CT numbers (combined different CaSupp indices with measured locations) and ADC values.

**TABLE 2.** Means and standard deviations of MRI apparent diffusion coefficient (ADC) and CT numbers in virtual calcium subtraction (VNCa) images for all measured locations

	**L1**	**L2**	**L3**	**L4**	**L5**	**Averaged L1−L5**
ADC	554.12±177.42	520.02±171.74	546.19±179.75	523.20±175.14	524.3±173.17	536.30±163.93
CaSupp 25	−236.58±77.07	−227.60±73.00	−217.83±83.35	−225.40±82.36	−244.67±79.27	−221.19±78.89
CaSupp 35	−138.15±46.58	−131.15±43.86	−127.11±51.4	−133.38±49.30	−143.10±48.23	−128.84±47.37
CaSupp 45	−82.04±30.19	−77.33±28.86	−75.38±34.45	−81.01±31.32	−85.24±31.31	−76.42±30.10
CaSupp 55	−45.12±20.87	−41.925±20.70	−41.32±24.80	−46.57±20.95	−47.08±21.44	−41.92±19.91
CaSupp 65	−18.50±16.35	−16.38±17.05	−16.79±19.89	−21.71±15.83	−19.71±16.14	−17.07±14.58
CaSupp 75	2.03±15.39	3.29±16.53	2.12±18.3	−2.58±14.9	1.42±14.54	2.08±13.26
CaSupp 85	18.63±16.60	19.22±17.75	17.45±18.93	12.93±16.52	18.55±15.37	17.61±14.59
CaSupp 95	32.46±19.15	32.46±20.12	30.25±20.75	25.98±19.16	33.00±17.41	30.59±17.20

CaSupp = calcium-suppressed index

### Characterize tumor burden with optimal combination of CaSupp index and vertebral body

**FIGURE 4.**
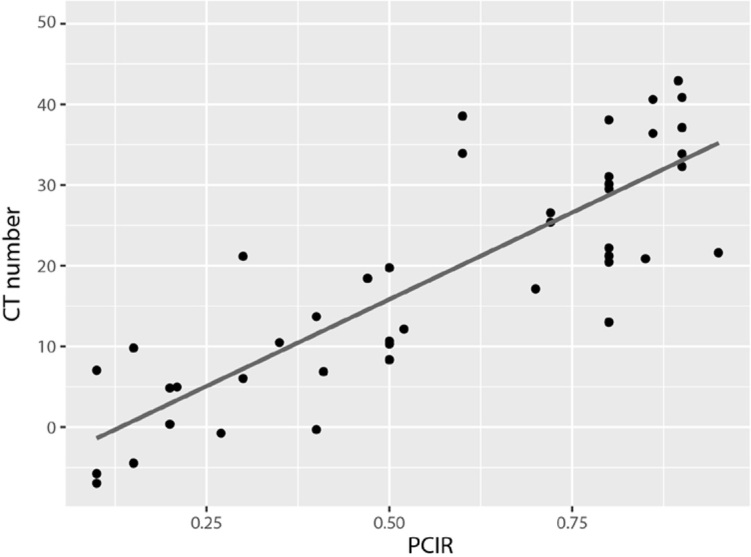
Bivariate correlation between CT number (averaged L1−L5 at calcium-suppressed [CaSupp] index 85) and plasma cell infiltration rate (PCIR) confirmed by bone marrow biopsy. The Pearson’s r yields 0.835 with p value < 0.001.

The CT number of averaged L1−L5 at CaSupp index 85 showed significant correlation with the PCIR (r = 0.835, p < 0.001) confirmed by BM biopsy (Figure 4). It showed a mean infiltration ratio of 54% (range, 10%−95%; median 60%).

**FIGURE 5.**
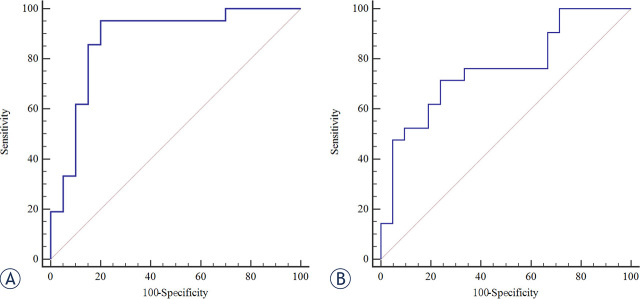
Receiver operating characteristic curves for CT number (averaged L1−L5 at calcium-suppressed [CaSupp] index 85) to predict “high serum-free light chains (SFLC) ratio” **(A)** and “cytogenetic status” **(B)**.

We performed ROC analysis with the predictor binary outcome “SFLC ratio” and “cytogenetic status” using the CT number of averaged L1−L5 at CaSupp index 85. Expectedly, it exhibited satisfying performance for discriminating high and low SFLC ratio with area under the curve (AUC) of 0.876 (0.736−0.958). The corresponding sensitivity, specificity and cutoff value were 0.952, 0.800, 10.66, respectively. Also, AUC for prediction of the “cytogenetic status” was 0.760 (0.603−0.878). The corresponding sensitivity, specificity and cutoff value were 0.714, 0.762, 20.43, respectively (Figure 5A, B).

## Discussion

Our results showed that VNCa images derived from DLCT could estimate BM infiltration with MRI as the reference, especially the CT number of averaged L1−L5 at CaSupp index 85 showed the highest correlation with ADC. What’s more, it allowed quantitative evaluation of tumor burden by correlating with PCIR and anticipating high SFLC ratio and the HRC status.

Instead of activating a second X-ray tube or rapid-voltage switching tube before performing the examination, DLCT adopts two different detector layers to decrease X-ray dose. For postprocessing, the flexible vendor could construct different parameters. VNCa image is a common parameter in musculoskeletal system^9,22,23^, in which the osseous component is removed from the spectral base data in order to improve visualization of BM. The degree of calcium suppression depends on the CaSupp index, which defines the calcium composition level. Several documents have confirmed the importance of VNCa image. Fervers *et al*. assumed that the pathologic BM was defined as voxels >0 HU and concluded that it could significantly predict BM infiltration, osteolytic lesions and the clinical diagnosis of MM.^14^ However, there is no consensus for the CT cutoff number of pathologic BM. Brandelik *et al*. assessed the potential of VNCa images to reflect BM infiltration.^16^ They evaluated the different regions (C7, T12, L1−L5) and infiltration patterns (non-diffuse and diffuse). However, C7 is not the typical region for BM infiltration and could be influenced by beam hardening artifacts easily as far as we know.^24^ Fervers *et al*. also investigated if VNCa images might discriminate metabolically vital, focal lesions from avital lesions in MM patients with FDG PET/CT as the standard of reference.^15^ Best result was yielded by high calcium suppression, followed by medium and low calcium suppression. However, the median interval time between DECT and FDG PET/CT was 53 days which was so long to leave time window for possible change in tumor biology between two images. In our study, the CaSupp indices ranged from 25 to 95 with an interval of 10 to search for the optimal CaSupp index, which may be more scientific and comprehensive. There is a growing tendency of the importance for increased CaSupp index that high CaSupp index could provide more information for BM infiltration and tumor burden than low CaSupp index. This might due to gradual exposure of underlying plasma cell cluster by increasing calcium suppression, which further validates VNCa images as a measurement tool for tumor burden. The averaged L1−L5 seems to be more representative than single lumbar vertebra due to the large size of those vertebrae with maximized reliable measurement avoiding sclerosis, fractures, or disc herniations. We did not divide the infiltration pattern according to MRI performance and we believe that this “agnostic” approach provides a more reliable marrow sample for evaluation of BM infiltration.^25^

We have included laboratory biomarkers to evaluate MM tumor burden. Among them, PCIR was obtained through BM biopsy on the iliac crest clinically, which is painful and uncomfortable for most patients. Despite IMWG recommendation^26^, a recent large-scale clinical analysis was performed to explore whether BM biopsy is necessary in all patients diagnosed with monoclonal protein since in some cases it did not contribute to the diagnosis. In our study, PCIR was correlated well with CT number of averaged L1−L5 at CaSupp index 85. Thus, it’s promising to obtain PCIR results by measuring CT number noninvasively. Due to different thresholds for the serum paraproteins of MM subtypes (e.g., IgA, IgG, IgM), only SFLC ratio was taken into consideration which is also an important indicator of tumor burden. In 2014, the IMWG included the SFLC ratio in the diagnostic criteria for MM, and SFLC ratio >100 is considered as a biomarker for ultrahigh-risk smoldering MM patient.^4^ However, some MM patients are non-secretory or hypo-secretory and are therefore difficult to surveille by means of serologic/urine markers alone which influences patient management at primary diagnosis and during therapy.^27^ What’s more, myeloma may escape hematologic diagnosis if it extends outside the marrow cavities (extramedullary).^28^ Similarly, ROC analysis indicates satisfactory performance for VNCa images to discriminate high and low SFLC ratio with AUC 0.876. Some studies have found that CAs are significantly associated with the proliferation and secretion of tumor cells.^29,30,31^ It was obtained through different invasive methods such as FISH. However, this technique suffers some drawbacks. For example, the patients may experience the pain of biopsy and bear the expensive expenses. What’s more, the BM results may be influenced by intratumoral heterogeneity and poor sample quality.^32–33^ So, developing a convenient and noninvasive method to predict cytogenetic status is critical for clinicians and patients. The results showed that VNCa images could anticipate HRC status with preferable AUC, sensitivity and specificity of 0.760, 0.714 and 0.762. Since the above specific situations may exist in clinical practice, such as painful and unbearable biopsy for some patients, non-secretory or hyposecretory M protein, extramedullary infiltration et al., DLCT could be employed to evaluate tumor burden additionally.

There are some limitations that needed to be discussed. First, the number of patients was rather small. Since the incidence rate of MM is lower than other diseases and is complex to deal with, so patients are usually admitted to specialized hospitals. Second, it was validated in the lumbar vertebra which were considered as the representative region of BM infiltration and minimally affected by the intrauterine device. But this needs to be upscaled across the body and also has more robust measurement of technique accuracy. Third, correlation with PCIR was possible only for the pelvic bones, but this reflects the deficit of daily practice. Finally, this study investigated the ability of DLCT acquired by specific scanner, imaging protocols, and post-processing tools which may not widely applied in other institutions. In the future, more studies are needed for definitive evaluation of this powerful technological equipment.

## Conclusions

Quantitative assessment of VNCa images in DLCT is a potential determination of BM infiltration extent in MM for radiologists and would be promising incorporated into the daily clinical practice, especially when the gold standard MRI is not accessible. Therefore, VNCa images could be used as additional imaging biomarkers for non-invasive assessment of tumor burden.
